# The distinct effects of metformin and imeglimin on high glucose-induced alterations in metabolic function and reactive oxygen species production in mouse Schwann cells are modulated by pemafibrate and/or fatty acid-binding proteins.

**DOI:** 10.3389/fncel.2025.1634262

**Published:** 2025-08-22

**Authors:** Hiroshi Ohguro, Megumi Higashide, Nami Nishikiori, Toshifumi Ogawa, Masato Furuhashi, Tatsuya Sato, Megumi Watanabe

**Affiliations:** ^1^Department of Ophthalmology, Sapporo Medical University School of Medicine, Sapporo, Japan; ^2^Division of Cellular Physiology and Signal Transduction, Department of Physiology, Sapporo Medical University School of Medicine, Sapporo, Japan; ^3^Division of Cardiovascular-Kidney-Metabolic Medicine, Department of Internal Medicine, Sapporo Medical University School of Medicine, Sapporo, Japan

**Keywords:** Schwann cells, metformin, imeglimin, FABP5, FABP7, extracellular flux analyzer, ROS

## Abstract

**Background:**

Imeglimin (Ime), the first in a novel class of antidiabetic agents, has potential therapeutic effects on diabetic peripheral neuropathy (DPN). This study aimed to evaluate and compare the effects on cellular metabolic function and reactive oxygen species (ROS) levels in high glucose-treated mouse Schwann cells (SCs), an *in vitro* DPN model, with those of metformin (Met), a conventional antidiabetic agent known for its beneficial effects on DPN. The roles of PPARα and fatty acid-binding proteins 5 and 7 (FABP5 and FABP7), both of which have been implicated in the pathogenesis of DPN, were also investigated.

**Methods:**

Schwann cells were treated with high glucose, Ime, Met, a selective PPARα agonist pemafibrate (Pema), or a FABP5/FABP7 inhibitor (MF6). Cell viability assays, extracellular flux analysis, and ROS production assays were performed.

**Results:**

No significant changes in cell viability were observed with any treatment. High glucose exposure increased glycolytic reserve compared to normal glucose conditions. Ime increased mitochondrial respiratory functions, whereas Met suppressed mitochondrial respiration and enhanced glycolytic functions, with these effects being more evident under normal glucose conditions. Pema significantly increased basal glycolysis under high glucose conditions, while MF6 had no appreciable effect. Both Ime and Met reduced ROS production in high glucose-treated SCs, with Ime exhibiting a more potent effect. However, the ROS-reducing effects of Ime and Met were abolished by Pema or MF6.

**Conclusion:**

Imeglimin exerted beneficial biological effects by enhancing the energetic state and reducing ROS production without inducing metabolic quiescence in high glucose-treated SCs. These findings suggest that Ime has therapeutic potential for DPN, although its effects may be modulated by intracellular lipid metabolism.

## Introduction

Diabetic peripheral neuropathy (DPN), a major complication of diabetes mellitus (DM), affects approximately half of patients with DM (Sloan et al., 2021). DPN primarily impairs sensory nerve function, but also impacts autonomic and motor nerves (Selvarajah et al., 2019). Approximately 30% of patients with DPN experience painful symptoms; however, only a limited number of patients achieve adequate pain relief with current therapeutic strategies (Jensen et al., 2021). Metformin (Met), a first-line therapy for type 2 DM (T2DM), has been reported to alleviate neuropathic pain in various contexts, including chemotherapy (Hacimuftuoglu et al., 2020), osteoarthritis (Wang et al., 2019), post-surgery (Smith and Tran, 2019), and inflammation (Augusto et al., 2022), possibly via activation of AMP-activated protein kinase (AMPK). Supporting this mechanism, PPARα agonists have been shown to improve DPN through the PPARα-AMPK-PGC1α-eNOS axis in db/db mice (Cho et al., 2014; Kołodziejski et al., 2017). In addition, it has been reported that inhibition of fatty acid-binding protein 5 (FABP5), an intracellular fatty-acid binding protein that regulates lipid signaling and metabolism, may also ameliorate peripheral neuropathy (Warren et al., 2024). Despite these therapeutic potentials, current strategies have not resulted in satisfactory clinical outcomes in the treatment of DPN.

Schwann cells (SCs), glial cells of the peripheral nervous system, play essential roles in supporting the growth, maintenance and repair of peripheral nerves (Bosch-Queralt et al., 2023). Their biological functions have been shown to deteriorate under hyperglycemic conditions, leading to various nerve dysfunctions including reduced conduction velocity and axonal atrophy (Dyck and Giannini, 1996; Eckersley, 2002). Although the precise mechanisms are unclear, metabolic changes in SCs under hyperglycemic and/or hyperlipidemic conditions, such as oxidative stress via overproduction of reactive oxygen species (ROS) as well as activation of the polyol pathway, glycation of lipids and proteins, and modulation of protein kinase C activity, are likely to be involved in the pathogenesis of DPN (Mizukami et al., 2011; Obrosova et al., 2007; Song et al., 2003; Vincent et al., 2009; Zhu et al., 2023). Indeed, recent studies suggest that dyslipidemia associated with DM contributes to the development and progression of DPN (Obrosova et al., 2007; Vincent et al., 2009). Therefore, amelioration of both metabolic dysregulation and ROS overproduction in SCs may be a promising therapeutic strategy for DPN. To investigate DPN pathogenesis and explore therapeutic strategies, *in vitro* models using cultured SCs under high glucose conditions have been widely utilized (Jiang et al., 2020; Zhang et al., 2021).

Imeglimin (Ime), the first drug in a new tetrahydrotriazine class of oral antidiabetic drugs called “glimins”, is expected to overcome many of the limitations of current therapies for T2DM (Konkwo and Perry, 2021). Recent clinical studies in both Japanese and Caucasian patients with T2DM have demonstrated that Ime provides significant and durable antihyperglycemic effects with favorable safety and tolerability profiles (Dubourg et al., 2021; Reilhac et al., 2022). Although the biological effects of Ime on DPN have not been fully elucidated, Ime, but not Met, has been reported to reduce ROS production, decrease the number of damaged mitochondria, and normalize mitophagic activity in islet β-cells of db/db mice (Lachaux et al., 2020; Nishiyama et al., 2023). Thus, Ime may have therapeutic potentials against DPN via suppression of ROS production in SCs.

In the present study, we aimed to elucidate the effects of Ime on DPN by comparing the effects of Ime and Met on cellular metabolic functions and ROS generation in mouse SCs under high glucose conditions. We also examined whether a PPARα agonist pemafibrate (Pema) or a FABP5 inhibitor MF6 modulates these actions under the same conditions.

## Materials and methods

### Planar cell culture of mouse SCs

All experiments were approved by the internal review board of Sapporo Medical University. Commercially available mouse Schwann cells (SCs, Catalog No. SWN-IMS32C, Cosmo Bio CO. Ltd., Tokyo, Japan) were cultured in planar culture dishes (diameter: 150 mm) until 90% confluence in a normal-glucose (5.5 mM)-DMEM supplemented with 10% FBS, 1% L-glutamine and 1% antibiotic-antimycotic and were maintained by daily changing the medium under standard normoxia conditions (37°C, 5% CO_2_).

### Cell viability assay

Cell viability was determined using a commercially available kit (Cell Counting Kit-8, Dojindo, Tokyo, Japan), as previously described (Pan et al., 2017). Briefly, SCs were incubated with 10 μl of a reactive solution for 2 h and then absorbance at 450 nm was measured using a microplate reader (multimode plate reader EnSpire^®^, PerkinElmer, MA U.S.A.) to determine cell viability.

### Measurement of real-time cellular metabolic functions

Under various conditions, the rates of oxygen consumption (OCR) and extracellular acidification (ECAR) of SCs were determined by using a Seahorse XFe96 Bioanalyzer (Agilent Technologies, Santa Clara, CA, U.S.A.) as described previously with slight modification (Higashide et al., 2024). Briefly, 20 × 10^3^ SCs pre-cultured in media containing different glucose concentrations (5.5 mM and 50 mM) for 24 h were seeded into each well of an XFe96 Cell Culture Microplate (#103794-100, Agilent Technologies, Santa Clara, CA, USA) and they were further treated with (1) dimethyl sulfoxide (DMSO) as a control, (2) 2 mM Met, (3) 2 mM Ime, (4) 10 μM pemafibrate (Pema), and (5) 10 μM MF6 for 24 h. On the assay day, the culture medium was replaced with Seahorse XF DMEM assay medium (pH 7.4, containing 5.5 mM glucose, 2.0 mM glutamine, and 1.0 mM pyruvate), followed by incubation in a CO_2_-free incubator at 37°C for 30 min. OCR and ECAR were simultaneously measured at baseline and after sequential injections of oligomycin (2.0 μM), FCCP (5.0 μM), and rotenone/antimycin A mixture (1.0 μM). Values of OCR and ECAR measured were normalized by the amount of protein per well. Protein concentration measured by the BCA protein assay (TaKaRa Bio, Shiga, Japan).

Various metabolic indices including basal respiration, ATP-linked respiration, proton leak, maximal respiration, Non-mitochondrial respiration, basal ECAR, glycolytic reserve, non-glycolytic acidification and baseline OCR/ECAR ratio were determined as described previously (Higashide et al., 2024).

### Measurement of levels ROS

Levels of reactive oxygen species (ROS) in SCs were measured by a Dichlorofluorescin-diacetate (DCFH-DA) method using a commercially available ROS assay kit (DOJINDO, Kumamoto, Japan) (Okazaki et al., 2019). SCs seeded in a 96-well clear bottom black plate (Greiner Bio-One, Austria) were cultured in a medium with different glucose concentrations (5.5 mM, 25 mM, and 50 mM) for 24 h. SCs were further treated with (1) dimethyl sulfoxide (DMSO) as a control, (2) 2 mM Met, (3) 2 mM Ime, (4) 10 μM pemafibrate (Pema), (5) 10 μM MF6, (6) various combinations of 2 mM Met or 2 mM Ime with 10 μM Pema and/or 10 μM MF6 for 24 h.

Cells were washed twice with Hanks’ Balanced Salt Solution (HBSS), suspended in 100 μl of Highly Sensitive DCFH-DA Working Solution and incubated for 30 min at 37°C, equilibrated with 95% air and 5% CO_2_. For evaluation of levels of ROS, fluorescence intensities were measured using a fluorescence plate reader (Excitation: 490 nm, Emission: 530 nm) after washing twice and suspended with HBSS to assess intracellular ROS levels.

### Gene expression analyses

After total RNA extraction, reverse transcription and quantitative real-time PCR (qRT-PCR) were processed as previously reported (Ida et al., 2020; Itoh et al., 2020) using specific primers and probes (Supplementary Table 1).

### Statistical analysis

For statistical estimation, one-way ANOVA followed by Tukey’s HSD (Honestly Significant Difference) *post hoc* analysis was carried out using Graph Pad Prism version 9 (GraphPad Software, San Diego, CA) as described in our recent reports (Ida et al., 2020; Itoh et al., 2020). The analysis was conducted under the assumption of normality, which is commonly applied in similar experimental settings. A *p* value less than 0.05 was statistically significant as indicated by asterisks.

## Results

To study the effects of antidiabetic or lipid metabolism modulating including Met and Ime, Pema and MF6 on DPN pathogenesis including metabolic derangements and oxidative stress, high glucose-stimulated SCs were used as an *in vitro* model mimicking pathogenesis of DPN. In this study, we adopted a concentration of 0.5 mM or 2 mM for both Met and Ime, which exceeds their clinically observed plasma levels (Chevalier et al., 2023; Kajbaf et al., 2016). This dosage was selected based on previous reports demonstrating dose-dependent effects on AMPK activity and mitochondrial function at concentrations ranging from 0.25 to 10 mM in various cell types (Hozumi et al., 2023). Notably, 0.5–2 mM is commonly used for evaluating mitochondrial responses *in vitro* (Hallakou-Bozec et al., 2021; Hozumi et al., 2023; Takahashi et al., 2024).

**FIGURE 1:**
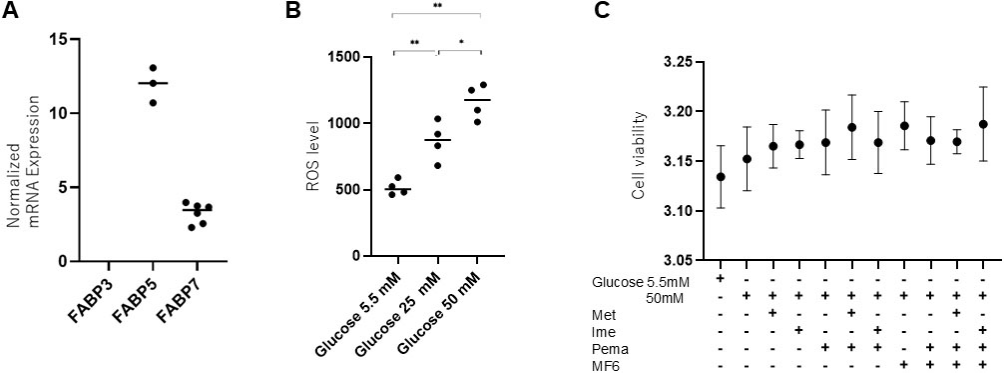
Expression of FABP isoforms and the impact of glucose concentrations and pharmacological metabolic modulators in SCs. **(A)** To verify the presence of mRNA expression of FABP3, FABP5 or FABP7, qPCR analysis was conducted. Experiments were performed in duplicate or triplicate using freshly prepared cells (*n* = 3–6). Expression levels of FABP3, FABP5, and FABP7 were normalized to the expression of internal control 36B4 (Rplp0). **(B)** ROS levels were examined under different glucose concentrations (5.5 mM, 25 mM, and 50 mM) in SCs (*n* = 4–5). **(C)** To evaluate the cytotoxicity of Met (2 mM), Ime (2 mM), Pema (10 μM) and MF6 (10 μM) on SCs under a normal-glucose condition (5.5 mM) or a high glucose condition (50 mM), cell viability was assessed by using a commercially available kit (*n* = 3). Data are presented as means ± the standard error of the mean (SEM). N.D., not detected. *p < 0.05, **p < 0.01.

First, we examined the types of FABPs expressed in mouse SCs and the glucose concentration-dependent ROS levels. As shown in Figure 1A, we confirmed positive expression of FABP5 and FABP7 among FABP3, FABP5 and FABP7, which are known to be related to the pathogenesis of DM and neuronal disorders (Boneva et al., 2011). FABP5 and FABP7 were also detected in SCs at protein levels (Supplementary Figure 1). Moreover, we found that ROS levels were increased in a glucose concentration-dependent manner in SCs (Figure 1B). Therefore, in the subsequent experiments, we adopted MF6, a specific inhibitor for FABP5 and FABP7, to inhibit the role of FABPs and selected 50 mM glucose as the high-glucose condition, considering it suitable for examining the effects of Ime, Met, and other agents in a more severe hyperglycemic state that mimics the pathogenesis of DPN. We also confirmed negligible levels of toxicity in SCs by (1) normal-glucose (5.5 mM) and high glucose (50 mM) conditions, (2) 2 mM Met, (3) 2 mM Ime, (4) 10 μM Pema and (5) 10 μM MF6 (Figure 1C).

**FIGURE 2:**
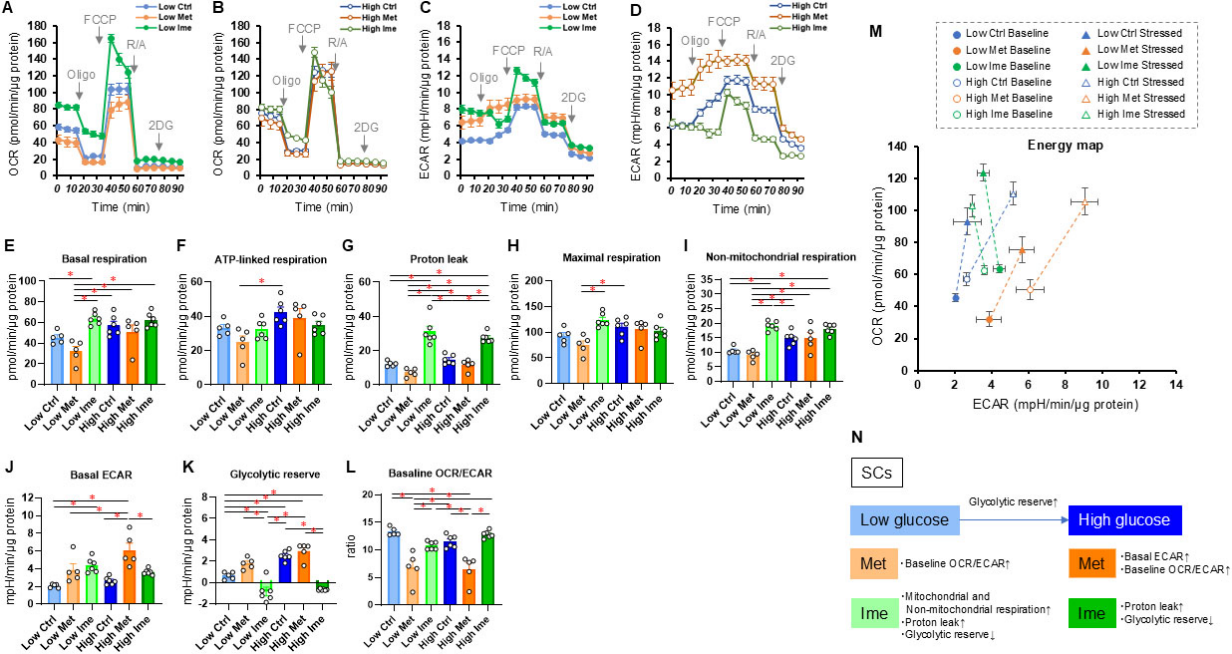
Effects of metformin (Met) or imeglimin (Ime) on cellular metabolic functions of SCs under normal-glucose and high glucose conditions. SCs were incubated for 24 h under normal-glucose (5.5 mM, Low) or high glucose (50 mM, High) conditions in the absence (Ctrl) or presence of 2 mM Met or 2 mM Ime. Then each specimen was subjected to a Seahorse extracellular flux analysis. **(A)** Plots of oxygen consumption rate (OCR) values in SCs that were treated under normal-glucose condition. **(B)** Plots of OCR values in SCs that were treated under high glucose condition. **(C)** Plots of extracellular acidification rate (ECAR) values in SCs that were treated under normal-glucose condition. **(D)** Plots of OCR values in SCs that were treated under high glucose condition. **(E–L)** Key metabolic indices. **(M)** Energy map of SCs in each group. The term “Baseline” refers to ECAR and OCR values at baseline, and the term “Stressed” refers to ECAR after administration of oligomycin and OCR after administration of FCCP. **(N)** Schematic summary of metabolic alteration induced by glucose concentration, Met or Ime. Data are presented as means ± the standard error of the mean (SEM). All experiments were performed using fresh preparations (*n* = 5–6). *P < 0.05.

Next, to address effects of antidiabetic agents, Met and Ime, on cellular metabolic functions of Schwann cells under a high glucose condition, we conducted extracellular flux analysis of SCs that were treated with Met and Ime under normal-glucose (5.5 mM) and high glucose (50 mM) conditions, respectively (Figures 2A–D). SCs treated with high glucose showed no statistically significant changes in metabolic parameter except for an increase in glycolytic reserve, yet there was a tendency toward enhanced metabolic activity with high glucose treatment (Figures 2E–K). Treatment with Met increased glycolytic functions and decreased baseline OCR/ECAR ratio (Figure 2L), but interestingly, treatment with Ime rather enhanced mitochondrial respiratory functions (Figures 2E–I). Such metabolic changes were more evident in SCs that were treated with normal-glucose condition compared those with high glucose condition (Figures 2E–I). Ime also increased proton leak regardless of glucose concentrations, whereas Met did not affect proton leak (Figure 2G). The findings suggest that Ime, as opposed to Met, enhances most of mitochondrial respiratory functions in SCs (Figures 2M, N).

**FIGURE 3:**
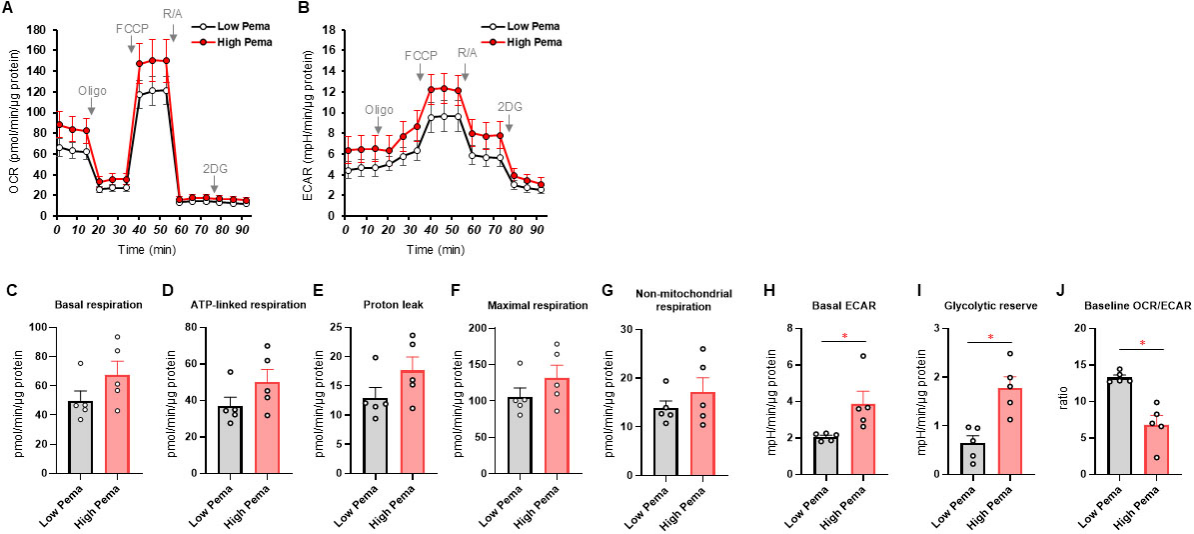
Effects of pemafibrate (Pema) on cellular metabolic functions of SCs under normal-glucose and high glucose conditions. SCs were incubated for 24 h under normal-glucose (5.5 mM, Low) or high glucose (50 mM, High) conditions in the presence of 10 μM Pema. Then each specimen was subjected to a Seahorse extracellular flux analysis. **(A)** Plots of oxygen consumption rate (OCR) values. **(B)** Plots of extracellular acidification rate (ECAR) values. **(C–J)** Key metabolic indices. Data are presented as means ± the standard error of the mean (SEM). All experiments were performed using fresh preparations (*n* = 5). *P < 0.05.

**FIGURE 4:**
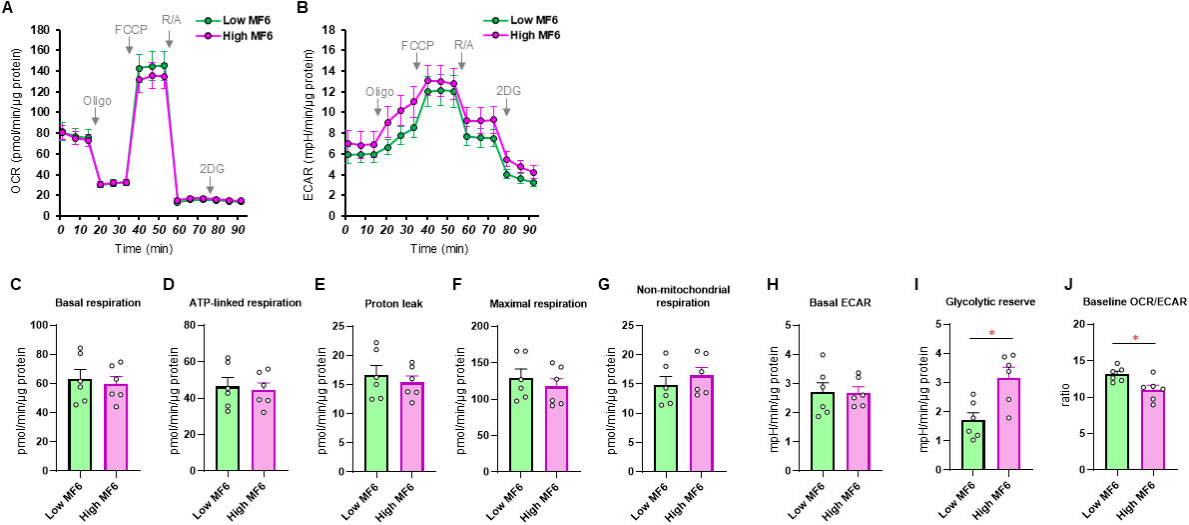
Effects of MF6 on cellular metabolic functions of SCs under normal-glucose and high glucose conditions. SCs were incubated for 24 h under normal-glucose (5.5 mM, Low) or high glucose condition (50 mM, High) conditions in the presence of 10 μM MF6. Then each sample was subjected to a Seahorse metabolic function analysis. **(A)** Plots of oxygen consumption rate (OCR) values. **(B)** Plots of extracellular acidification rate (ECAR) values. **(C–J)** Key metabolic indices. Data are presented as means ± the standard error of the mean (SEM). All experiments were performed using fresh preparations (*n* = 6). *P < 0.05.

Since it has been well-recognized that lipid metabolism derangements were also associated with the pathophysiology of the development of DPN, we next examined the effects of Pema (Figures 3A, B) and MF6 (Figures 4A, B) on metabolic functions in SCs that were cultured with normal-glucose and high glucose conditions. Pema tended to increase cellular metabolism toward energetic in the high-glucose condition, but the effects were more evident in glycolytic indices than mitochondrial respiratory indices (Figures 3C–J). In contrast, MF6 did not affect glucose dependent metabolic alterations albeit SCs expressed both FABP5 and FABP7 (Figures 4C–J). These findings suggest that PPARα-related signaling, but not FABP5/7-related signaling, may have influence high glucose-dependent metabolic alteration.

**FIGURE 5:**
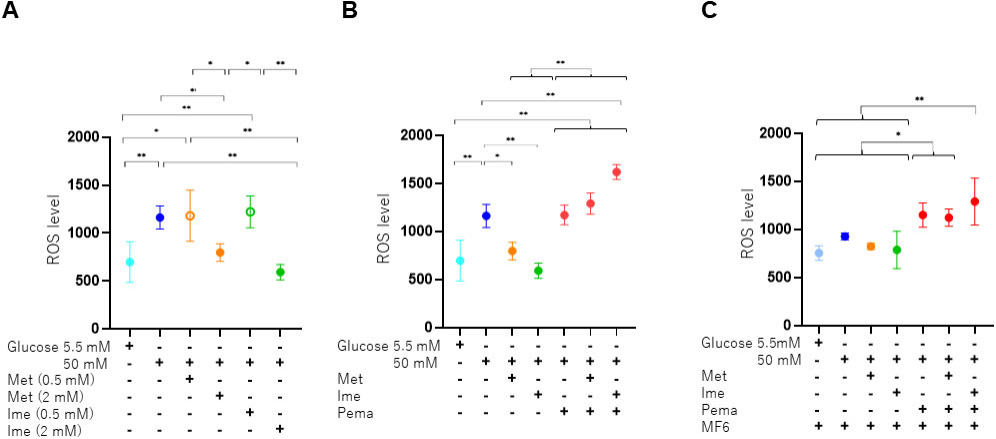
Levels of production of ROS in SCs under various conditions. SCs were incubated for 24 h under normal-glucose (5.5 mM, Low) or high glucose (50 mM, High) conditions in the absence or presence of various combinations of 0.5 or 2 mM Ime, 0.5 or 2 mM Met, 10 μM Pema or 10 μM MF6. The SCs were then subjected to measurement of levels of ROS, and those values were plotted. **(A)** Met or Ime under a normal-glucose or high glucose condition, **(B)** Met or Ime in the presence of Pema under a high glucose condition, and **(C)** Met, Ime and/or Pema in the presence of MF6 under a high glucose condition. All experiments were performed in triplicate using fresh preparations (*n* = 4). Data are presented as means ± standard error of the mean (SEM). *p < 0.05, **p < 0.01.

To study the effects of Ime, Met, Pema and/or FABPs on high glucose-induced oxidative stress of SCs, levels of ROS were measured. As shown in Figure 5A, (1) levels of ROS were significantly increased under a high glucose condition compared to those under a normal-glucose condition and (2) such elevated levels of ROS were reduced to varying degrees by 2 mM Met and Ime but not by 0.5 mM of those. The effects of 2 mM Ime were more potent than those of 2 mM Met. We also evaluated the combined effects of 2 mM Met or Ime with Pema and/or MF6 under a high glucose condition. As shown in Figures 5B, C, (1) Pema significantly increased the generation of ROS regardless of the conditions with or without Met or Ime and (2) MF6 substantially reduced the suppressive effects of Met and Ime on generation of ROS. Therefore, taken together, the results indicated that Ime had a more potent inhibitory effect than that of Met on high glucose-induced generation of ROS in SCs and the beneficial effects were greatly influenced by FABPs and PPARα-related signaling.

Finally, to explore the potential mechanisms by which Ime or Met suppresses high glucose-induced ROS production in SCs, we assessed the expression of hypoxia-related genes including HIF1A and VEGFA, which are closely associated with regulation of cellular metabolism and ROS production (Majmundar et al., 2010). Although the expression levels of these genes were decreased in a high glucose condition compared with a normal glucose condition in SCs, there were no statistically significant difference between Ime-treated group and Met-treated group under a high glucose condition (Supplementary Figure 2). These findings suggest that suppression against high glucose-induced ROS production by Ime or Met are independent of hypoxia inducible factor-related signaling.

**FIGURE 6:**
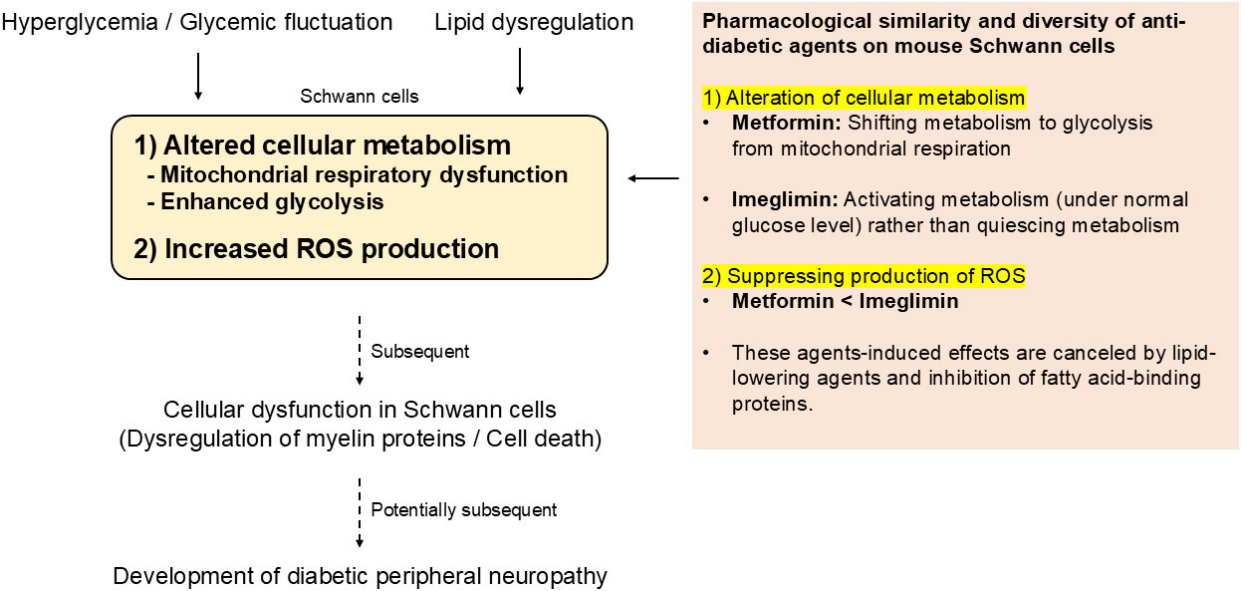
Overview of the present study and perspective for possible roles of Ime in preventing DPN.

A schematic summary of the present study is shown in Figure 6.

## Discussion

It has been shown that elevated levels of glucose induce oxidative stress in neuronal cells and Schwann cells (Askwith et al., 2009; Khan et al., 2023; Obrosova et al., 2005; Schmeichel et al., 2003) and that increased levels of ROS harm the lipids in myelinated nerves, leading to the loss of axons and destruction of the microvasculature in the peripheral nervous system (Vinik et al., 2006). As for the possible underlying mechanisms causing the hyperglycemia-induced oxidative stress, it was suggested that the persistent hyperglycemic state in T2DM could modulate mitochondrial DNA (mtDNA) and nuclear DNA (nDNA) (Fernyhough et al., 2003), thereby inducing mutations in the mitochondrial genome as well as mitochondrial dysfunction to produce ROS (Hollensworth et al., 2000; Srinivasan et al., 2000). In the present study, a high glucose condition caused significant increases in the levels of ROS and glycolytic reserve in SCs. In addition, the high glucose-induced increase in the levels of ROS was substantially reduced by Met and Ime and the effect of Ime was much more potent than that of Met. Since previous studies showed that Met had beneficial effects on diseases of the peripheral nervous system including DPN by inhibition of the harmful effects of ROS on cell number, cell viability, and migration (Ma et al., 2015), the present study suggested that Ime may have more potent beneficial effect than that of Met on DPN.

Intriguingly, while Met decreased mitochondrial respiratory functions, Ime rather increased indices of mitochondrial respiratory functions in SCs, especially in a normal glucose condition. In addition, Ime increased proton leak irrespective of low- or high-glucose exposure (Figure 2G). Given that Ime suppressed high glucose-induced ROS production in SCs (Figure 4), such increase in proton leak may be associated with ROS suppression via activating of uncoupling proteins (Mailloux and Harper, 2011), although there seems no significant increase in proton leak by Ime in other types of cells including hepatocytes or pancreatic islet β-cells (Hozumi et al., 2023; Li et al., 2022). Indeed, previous reports have shown that Ime increases levels of nicotinamide adenine dinucleotide (NAD^+^) (Hallakou-Bozec et al., 2021; Takahashi et al., 2024), which is a potentially positive regulator of uncoupler proteins through NAD^+^-Sirt1/AMPK-PGC-1α pathway (Nishikiori et al., 2025; Xu et al., 2021). Furthermore, we evaluated the expression levels of representative mitochondrial respiratory chain complexes in SCs in both low- and high-glucose conditions with and without Ime, but there were no remarkable differences in these protein levels (Supplementary Figure 3). Therefore, the changes in cellular metabolism by Ime observed in the present study are likely to be due to the activation of whole metabolic pathways, or changes in the intermittent metabolites, rather than changes in the expression or activities of the mitochondrial respiratory chain complexes themselves in SCs. Future comprehensive analysis may clarify these unresolved molecular mechanisms. Nevertheless, it is noteworthy that Ime activated cellular metabolism in SCs without increasing ROS production, whereas activation of cellular metabolism is generally associated with ROS production. This comprehensive finding supports the idea that Ime may ameliorate disease states in which increased ROS is the main pathophysiology. Although further clinical trials are needed to determine whether Ime significantly reduces the incidence of DPN compared to other anti-diabetes agents, the present findings support the recent notion that the choice of anti-diabetes agent should not focus solely on lowering blood glucose levels, but should also consider its protective effects on various organs (Qaseem et al., 2024).

It has been shown that dyslipidemia causes vascular insufficiency, oxidative stress-induced deterioration of mitochondrial function, and impaired conduction of electrical impulses in neurons, which are involved in the onset and progression of DPN (Davis et al., 2008; Wiggin et al., 2009), and normalization of lipid metabolism is therefore a suitable therapeutic strategy for DPN. A previous study showed that oral administration of the PPARα agonist fenofibrate, which is widely used to treat hyperlipidemia (Wierzbicki et al., 2003), prevented the progression of sciatic neuropathy in diabetic mice by activating of the AMPK-related signaling pathway (Cho et al., 2014). Furthermore, it has been shown that a PPARα agonist stimulated corneal nerve regeneration in patients with T2DM (Teo et al., 2023) and protected the corneal nerve from degeneration in a mouse model of diabetes (Matlock et al., 2020), suggesting that the use of the PPARα agonist may be promising strategy for treating DPN. In the present study, although the PPARα agonist Pema increased both mitochondrial and glycolytic functions under a high glucose condition, only Pema induced a significant increase in the levels of ROS and the Pema-induced elevation in the levels of ROS was synergistically enhanced by Met and Ime. Similar to this, a recent study showed that bezafibrate improved mitochondrial morphology and functions despite causing a mild increase in the production of ROS using fibroblasts obtained from patients with dynamin-1-like protein (DNM1L) mutation (Douiev et al., 2020). In contrast, other studies showed that bezafibrate reduced the levels of ROS significantly in hiPSC-derived neural stem cells (NSC), early neural progenitors (eNP), and neural progenitors (NP) (Augustyniak et al., 2019) and that Pema inhibited angiotensin II-induced production of ROS in human vascular smooth muscle cells by increased catalase activity (Amioka et al., 2022). Furthermore, it was shown that fenofibrate could prevent DPN by protecting endothelial cells through VEGF-independent activation of the PPARα-AMPK-PGC-1α-eNOS-NO pathway (Cho et al., 2014). However, reason why effects of Pema on levels of ROS were different from other fibrates such as bezafibrate and fenofibrate remained to be elucidated. It was speculated that much higher efficacies of PPARα agonist activities and different off-target biological effects of Pema compared to other fibrates may be involved (Yamashita et al., 2020). Taken together, the results suggested that effects of the PPARα agonist Pema on production of ROS may be different among various experimental conditions using different sources of cells.

FABPs, which are known as intracellular lipid chaperones, were found to be expressed in cells of the central and peripheral nervous systems (De León et al., 1996; Furuhashi and Hotamisligil, 2008). Functionally, FABPs interact with various fatty acids and other endogenous lipids including endocannabinoids, thereby playing a pivotal role in the development of the nervous system as well as the mature nervous system to modulate neuronal activity and network functions (Lu and Mackie, 2021). Previous studies have shown that FABP5-deficient mice and mouse models with pharmacological inhibition of FABP5 showed an increase in brain levels of Anandamide (AEA) (Kaczocha et al., 2014; Yu et al., 2014). In contrast to FABP5, the contribution of FABP7 remains to be elucidated, although the expression of both FABP5 and FABP7 was observed in various nervous cells (Glaser et al., 2023; Kawahata and Fukunaga, 2023). However, it was shown that elevated levels of AEA were comparable in FABP5/7 KO mice and FABP5 KO mice (Kaczocha et al., 2014; Yu et al., 2014), suggesting that the FABP that is responsible for the regulation of the AEA levels may be FABP5 but not FABP7. In support of this, the expression level of FABP7 in the adult mouse brain is low (Owada et al., 1996). In the current study, we also detected the expression of both FABP5 and FABP7 in SCs and the expression level of FABP7 was lower than that of FABP5. In the present study, pharmacological inhibition of FABP5 and FABP7 by MF6 did not alter high glucose induced metabolic changes in SCs, whereas Ime- and/or Pema-induced effects on the levels of ROS production under high glucose condition, suggesting that FABP5 and FABP7 may stimulate high glucose induced oxidative stress in SCs independently of their metabolic states. In fact, a recent study showed that FABP5 caused mitochondrial dysfunction related to αSyn oligomerization/aggregation in mitochondria, thereby inducing oxidative stress in neurons (Wang et al., 2021).

The present study has several limitations. First, the precise molecular mechanisms by which Ime reduces oxidative stress in high glucose-treated SCs remain unclear. Although Ime alleviated oxidative stress under high-glucose conditions, this antioxidant effect occurred without concurrent improvement in mitochondrial respiration, suggesting the involvement of alternative pathways. Specifically, NAD^+^-related metabolism, including the role of nicotinamide phosphoribosyltransferase (NAMPT), is presumed to be the central molecular mechanism of action of Ime (Hallakou-Bozec et al., 2021). Elucidating these pathways through metabolomics, multiomics analyses including transcriptomics, and stable isotope tracer approaches across various cell types may further clarify the therapeutic potential of Ime. Second, while ROS levels were evaluated at both 0.5 mM and 2 mM concentrations of Met and Ime, the relevance of these concentrations to physiological or therapeutic levels remains uncertain. Further investigations are needed to validate whether similar effects occur under *in vivo* conditions. Third, we did not perform osmolarity equalization using agents such as sucrose or mannitol, as our aim was to mimic the pathophysiological hyperglycemic conditions observed in DM, where elevated glucose levels impose both metabolic and osmotic stress on cells. Finally, the findings in the present study do not directly demonstrate the efficacy of Ime in ameliorating DPN. In addition, the effects of Ime and Met on mitochondrial function have not been characterized in detail. Further studies are needed to clarify the additive roles of FABP and PPARα in Ime-induced responses by elucidating their downstream signaling and additional mitochondrial parameters, such as mitochondrial membrane potential in SCs. Moreover, *in vivo* studies using diabetic animal models are required to evaluate the effects of Ime on sensory disturbances and nerve conduction velocity.

## Conclusion

In conclusion, the antidiabetic agent Ime had a more potent suppressive effect than that of Met on a high glucose-induced *in vitro* model mimicking pathogenesis of DPN using mouse SCs and that their effects were exclusively and synergistically modulated by FABP or PPARα-related signaling. However, since all findings are obtained from *in vitro* models, the therapeutic potential of Ime for DPN should be interpreted with caution and requires further validation in *in vivo* studies.

## Data Availability

The raw data supporting the conclusions of this article will be made available by the authors, without undue reservation.
