# Supplementary figures and images for: The distinct effects of metformin and imeglimin on high glucose-induced alterations in metabolic function and reactive oxygen species production in mouse Schwann cells are modulated by pemafibrate and/or fatty acid-binding proteins
Source: Front Cell Neurosci. 2025 Aug 22;19:1634262. doi: 10.3389/fncel.2025.1634262 (PMC12411464; doi:10.3389/fncel.2025.1634262)

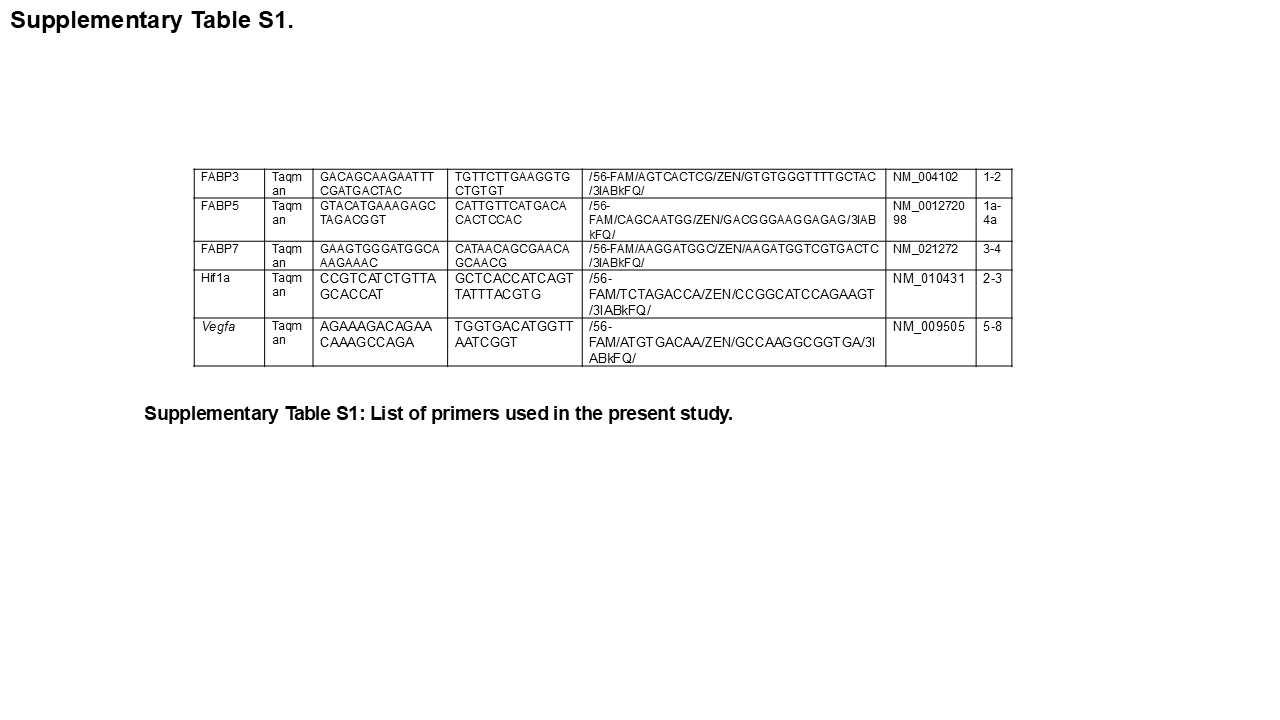

Supplement: Supplementary file 1 [file Image_1.tif]

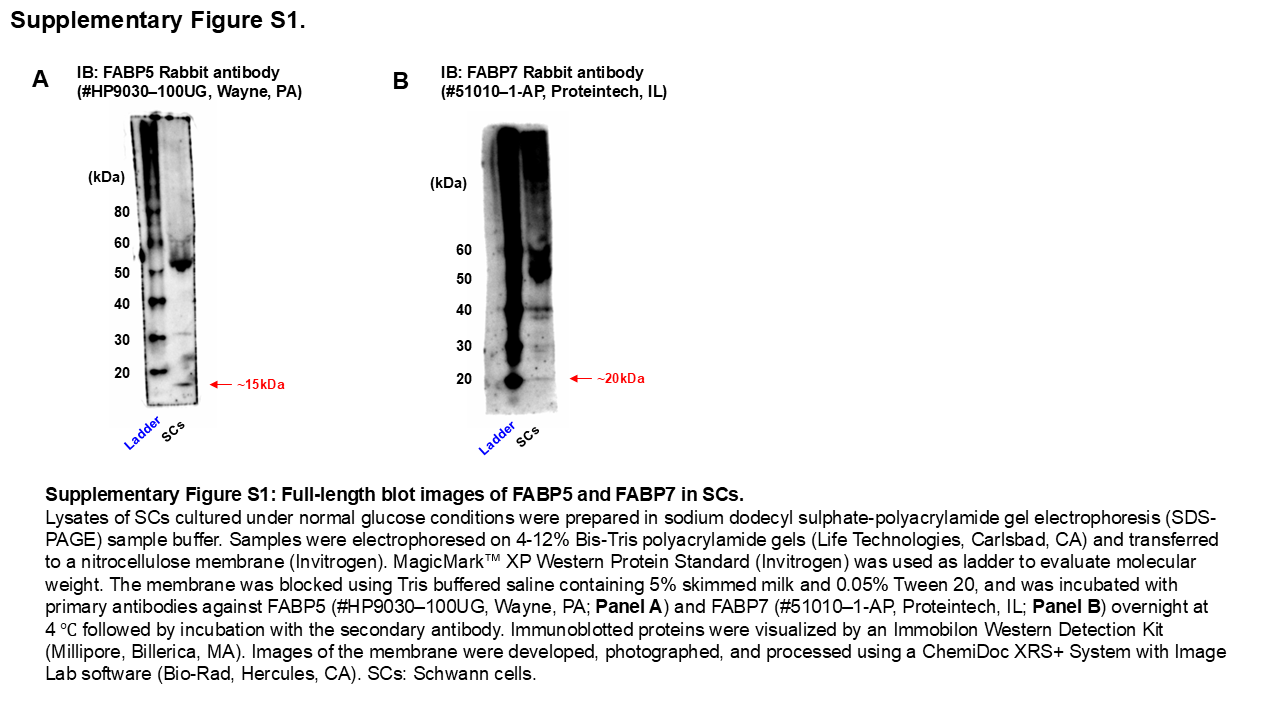

Supplement: Supplementary file 2 [file Image_2.tif]

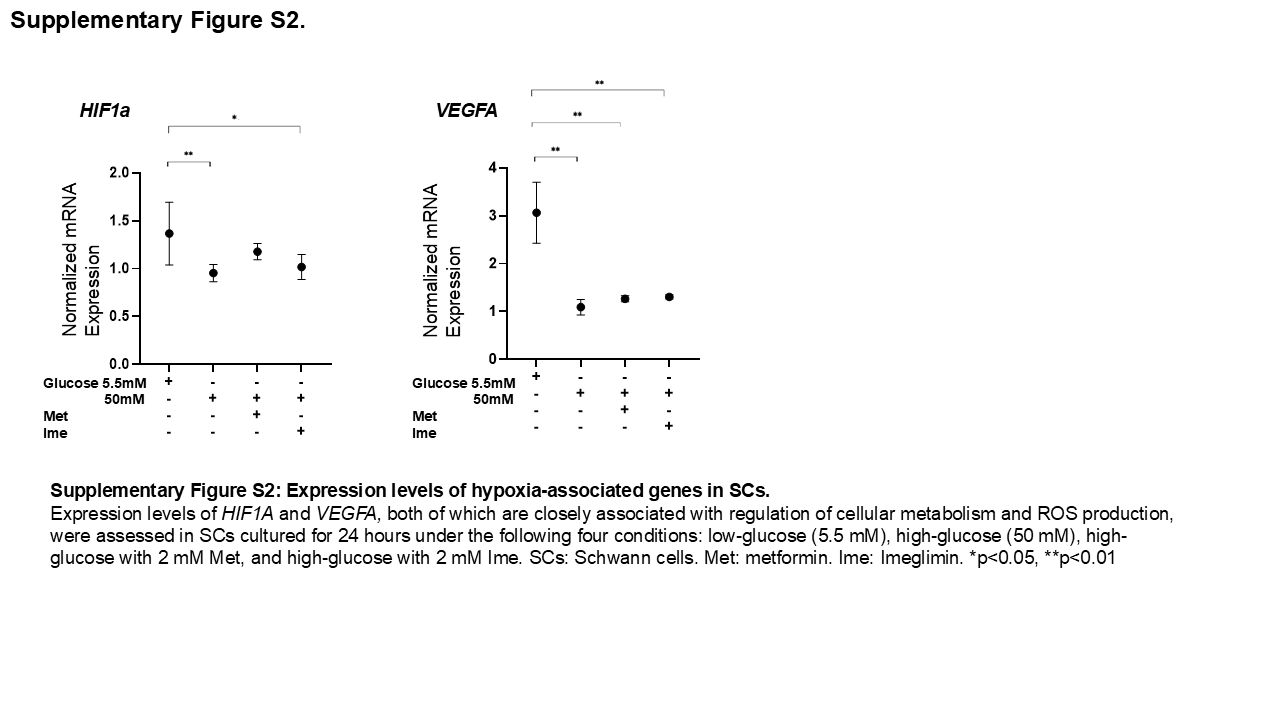

Supplement: Supplementary file 3 [file Image_3.tif]

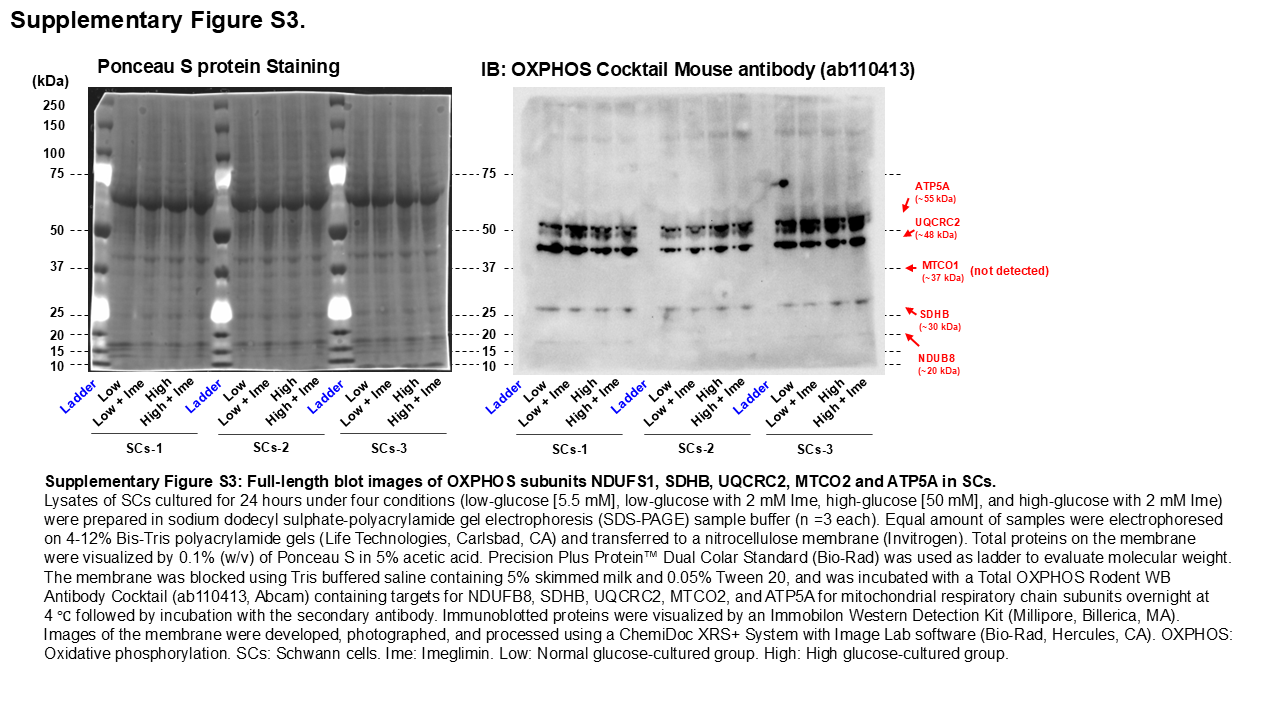

Supplement: Supplementary file 4 [file Image_4.tif]
